# The Limitations of Surgery

**Published:** 1903-07-04

**Authors:** 


### The Hospital.

A Journal of

The Medical Sciences and Hospital Administration.

Edited by Sir Henry Burdett, K.C.B.

Vol. XXXIV.—No. 875. [July 4, 1903.

### The Limitations of Surgery.

There has of late been an increasing amount of evidence, furnished by the acts and words of some of the most distinguished members of the profession of surgery, that they are beginning to view with a certain amount of distrust, or even with some anxiety, the growing practice of having recourse to the knife for the cure of a great variety of ailments, some of which, at least, might be amenable to other forms of treatment, while some might perhaps be endured without entailing any very serious consequences upon the sufferer. We lately heard it maintained by a very eminent surgeon, in reference to the amount of life which had been saved by the discoveries of Lord Lister, that there was at least some amount of loss to be placed on the other side, because, he said, "every fool now thinks that he may operate." The human mind is so constituted that it is more easy for most people to remember successes than failures, even if only by reason of the tendency to dwell somewhat fondly upon the former, while the latter, as far as possible, are pushed away out of sight and out of mind, or, if recalled, are used chiefly as stimulants to a hope for better luck next time. A certain proportion of the "early" operations for real or supposed "appendicitis" are at least of questionable necessity; and some of the surgeons who perform them are possibly but imperfectly acquainted with the clinical history of the malady as it presented itself to their fathers and grandfathers, or as it is described in the pages of such a work as Watson's *Lectures*. Some of them seem to share the popular delusion that appendicitis is a "new disease," and fail to recognise that it is but the old perityphlitis, writ, if not "large," at least with an attempt at greater precision. No doubt there are cases in which operative treatment affords the only hope of a successful issue, and there are surgeons fully capable of distinguishing these from others; but it is not with regard either to such cases or to such men that the question now before us arises. It has lately been brought into prominence by Sir William Bennett, K.C.V.O., the senior surgeon to St. George's Hospital, in a very admirable address on the "Ethics of Operations in Surgery," originally delivered before the Medical Society of London, and since revised and published in book form. The author refers especially to classes of cases in which he declares that the adoption of operative treatment has with some degenerated into routine, and he selects two illustrations as familiar ones, disease of the appendix and varix. The removal of the appendix after the occurrence, or recurrence, of certain symptoms may, he says, be described without exaggeration as a routine practice with many surgeons. At the same time, it cannot be contended either that the removal of the appendix is always called for, or that it is always followed by cure. He contends that an amount of judgment wholly incompatible with routine is demanded for the proper application of a treatment which may itself cause death, which may not relieve the symptoms for which it has been performed, and which sometimes gives rise to grave complications, such as extensive thrombosis. Into the subject of operations for the cure of varix we need hardly follow him.

If we look at the questions raised by Sir William Bennett from the standpoint of the general practitioner, who, in the great majority of cases, would be the person first called in, and who would be likely to exert considerable influence both as regards the choice of a consultant and as regards the course to be ultimately pursued, we must not forget that certain forms of operative treatment, the advantages or the necessity of which are now fully established, have only attained their present position after a long period of probation, often attended by a sufficient percentage of failure to cast grave doubts upon the general propriety of the proceeding. Ovariotomy is, of course, a salient example; and, if the pioneers of that operation had been less courageous, or less convinced, it would probably have been entirely abandoned. Between the two cases there is, however, this difference, that the pioneers of ovariotomy were few, and, as they almost monopolised the experience, so they gained in full measure the instruction which it was calculated to afford. Every unsuccessful case was as a beacon light to show dangers which might be provided against in the future. Moreover, there was no element of urgency in point of time. It was not necessary to decide on the instant. In actual or supposed disease of the appendix the operators are many, and the misadventures which may befall any one of them are not likely to be extensively known to the rest. The surgeon who desires to operate will usually, and from his point of view quite properly, protest against delay. He will say that now is the accepted time. In such circumstances it should, we think, be the province and the duty of the general practitioner to hold the balance of a skilled and an impartial judgment. He should bring his more intimate knowledge of the patient and his surroundings to bear upon the case, and should accept full responsibility for any advice which he may give. He should not allow himself to be "guided" by the opinion of a doubtless estimable gentleman, with a strong mental bias in some particular direction. In this way, and, we think, in this way only, will the public be likely to gain full security against what Sir William Bennett describes as routine surgery. "It would," he tells us, "be easy to indicate persons in the medical profession who, whilst they are the subjects of recurrent appendicitis, show no great anxiety for operation."

